# Impact of grape pomace on growth performance, carcass traits and meat colour in broiler chickens: Insights from a meta-analysis

**DOI:** 10.1016/j.psj.2025.105344

**Published:** 2025-05-29

**Authors:** Freddy Manyeula

**Affiliations:** Department of Animal Sciences, Faculty of Animal and Veterinary Sciences, Botswana University of Agriculture and Natural Resources, Gaborone, Botswana

**Keywords:** Broiler chickens, Carcass, Grape pomace, Meat colour, Phytochemical compounds

## Abstract

Grape pomace (**GP**) is a by-product from grape juice and wine production that is rich in potentially beneficial phytochemical compounds. Accordingly, several studies have been conducted to evaluate the effect of including GP in broiler chicken diets on a range of parameters that include growth performance, carcass traits, and meat quality. However, reported results have been inconsistent. Therefore, this meta-analysis investigates the effects of GP on Average daily feed intake (**ADFI**), average daily gain (**ADG**), feed conversion ratio (**FCR**), carcass traits, and meat quality in broiler chickens. The objective is to identify knowledge gaps and create new insights using published data. Twenty (20) research articles on the topic were identified via a systematic search done on selected online databases (Google scholar, Scopus, Web of Sciences, and PubMed) and thereafter, data were extracted and analyzed using OpenMEE software. A random‐effects model was used and presented as standardized mean difference (**SMD**) at a 95 % confidence interval (**CI**). Sources of heterogeneity were explored by subgroup and meta-regression analysis using moderators variables (broiler strains, inclusion levels, age, and sex). The results showed that dietary GP did not affect FI [SMD = -0.13; *P* < 0.001; *I^2^* = 89 %], ADG [SMD = -0.14; *P* < 0.001; *I^2^* = 80 %] and FCR [SMD = 0.00; *P* < 0.001; *I^2^* = 85 %] of broilers. Likewise, dressing percentage, breast, thigh, heart, and spleen weights in broiler chickens were not significantly affected. However, the weights of drumstick and gizzard were higher while liver weights were lower in broilers fed GP-based diets compared to those fed diets without GP. Regarding meat colour, broilers fed GP-based diets had higher meat redness compared to control. Meta-regression analysis revealed that broiler strains accounted for the most heterogeneity. In conclusion, dietary GP improved carcass traits and internal weight drumstick, gizzard weight, and meat redness in broiler chickens but had no effect on growth performance. Therefore, it is recommended that further investigation should be carried out to determine the optimal inclusion level of GP that support growth performance and liver weight in broiler chickens using optimisation model.

## Introduction

Poultry provide high quality animal protein to the global population thereby contributing to nutrition and food security of the country. Indeed, FAO (2012) predicted that the global population will be more than 9.2 billion by 2025 and that the total global food demand will increase by 35 to 56 % between 2010 and 2050 (Van Dijk et al., 2021). It is, therefore, very important for the poultry nutritionists to harness every available resources as feeding raw materials to improve the growth and outputs of poultry products and by-products. Additionally, the high cost of conventional feedstuffs is responsible for the continued high cost of production of poultry products, especially in developing countries. For example, the major conventional protein feed exploited in the poultry industry is mostly imported soybean which is known by its high quality protein and superior amino acids. Also, soybean is known to be unsustainable both economically and environmentally in drier region (Tamasgen et al., 2021). To address this issue, shift from disposal to recycling and upcycling could be a good strategies to reduce reliance on imported soybeans in countries where agronomic conditions are unfavorable for soybean production. Grapes are non climatic fruits, which are in high demand and consumed all-round the year in developing countries, there is a need to identify waste in this fruits that can assist in reducing pressure on soybeans and meet the demands of consumers for “clean,” natural’’ and green label products (Kasapidou et al., 2015) such as grape pomace (*Vitis vinifera*).

Grape pomace (GP) is a by-product of the production of grape juice and wine (Muhlack et al., 2018) which is ranked top five among all fruits globally (FAO, 2017). Although GP has no direct use as food for humans, its chemical characterization shows that it can be used in poultry production (Erincle et al., 2022). The GP contains flavonoids, which are known to have antioxidant capacity with the ability to prevent oxidative damage (Pandey and Rizvi, 2009) resulting in improvement in ADG (Aditya et al., 2018). Also, GP is known to contain fatty acids for modulating gastrointestinal tract microbiota and enhancing immunity and antibody titres levels (Thanabalan and Kiarie, 2021). The presence of proteins, carbohydrates, lipids, vitamins, minerals, and beneficial phytochemical such as polyphenols, phenolic acids and total flavonoids (Alfaia et al., 2022) makes GP a suitable candidate as poultry feedstuff. However, GP also contains total tannins and fibre which could impede absorption and binds nutrients (proteins and carbohydrates) leading to poor broiler health and growth when added at high inclusion levels (Chamorro et al., 2013).

In light of the above, studies have been conducted on the effect of GP on broiler chicken performance with inconsistent findings. Some authors found that GP had detrimental effects on broiler chicken productivity (Aditya et al., 2018; Erinle et al., 2022; Giamouri et al., 2022), while others reported beneficial effects on broiler performance (Gungor et al., 2021; Pop et al., 2015). This variation in results may be attributed to differences in broiler strains, growing phases, grape type, and inclusion levels, among others. Nonetheless, narrative literature cannot investigate how these factors could influence the utility of GP in broilers to derive with measurable assumptions of the efficiency of GP on broiler performances. Hence, there is a need to use statistical techniques to integrate published results on the topic to draw a stable and reliable scientific conclusion. Given the above, this review applies meta-analysis to systematically analyse growth performance, carcass and meat colour of broilers fed diets with GP. The meta-analysis was designed to answer the following research question: Does GP inclusion in the broiler diet affect growth performance, carcass traits and meat colour?.

## Material and methods

### Literature search, retrieval strategy, and coding

**Fig 1:**
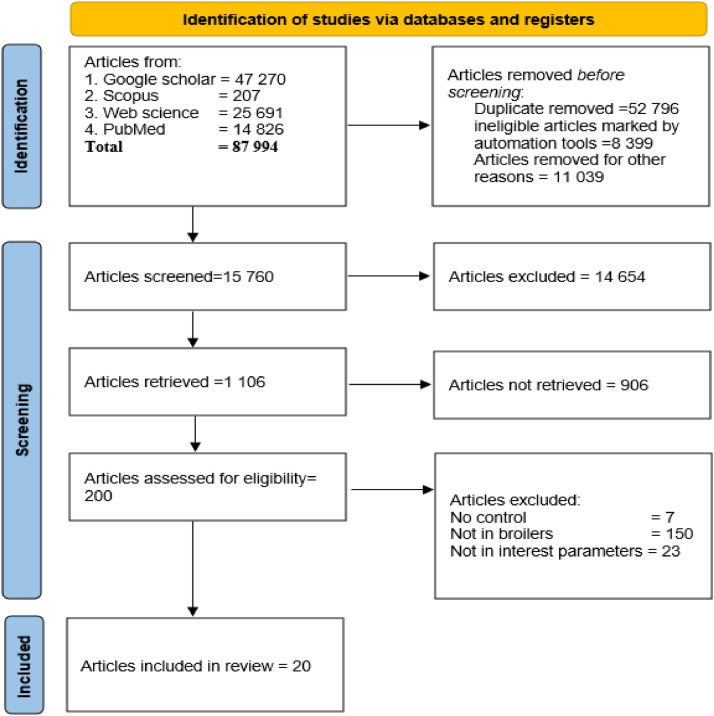
Literature search and selection process following the PRISMA procedure.

In this study, literature search was conducted in English databases such as Google Scholar (https://scholar.google.com), Scopus (https://www.scopus.com), Web of Sciences (https://www.webofscience.com), and PubMed (https://pubmed.ncbi.nlm.nih.gov) for relevant articles published from 2011 to 2024. The retrieval strategy is detailed in Table 1 and Fig. 1.

**Table 1:** A summary of a retrieval strategy used in this meta-analysis.

Database		Search words	Number
Google Scholar			
Search 1		Grape pomace NEAR broiler chickens	11,900
Search 2		Grape pomace WITHIN broiler chickens	15,300
Search 3		Grape pomace* broiler chicken*	4,770
Search 4		Grape pomace + broiler chickens	15,300
Scopus			
Search 1 from 2008 to 2024		Grape pomace AND broiler chicken	39
Search 2 from 2017 to 2024		"grape pomace" AND "broiler chickens"	24
Search 3		Grape pomace OR broiler chicken	123
Search 4 (2008-2024)		Grape pomace NOT broiler chicken	21
Web of Sciences			
Search 1		Grape pomace AND broiler chicken	19
Search 2		Grape pomace OR broiler chicken	24,748
Search 3		Grape pomace NOT broiler chicken	924
PubMed			
Search 1		Grape pomace OR broiler chicken	12 194
Search 2		Grape pomace NOT broiler chicken	2632

### Inclusion and exclusion criteria

Studies were selected using the PICO (Population, Intervention, Comparison, and Outcomes) framework (Table 2), thereafter the papers were screened using the following inclusion criteria; 1) study subjects: healthy commercial broiler chickens; 2) Research types: studies that report growth performance, carcass, and meat colour of broiler chickens in English and presented in mean value and standard deviation (SD) or standard error (SE) of the relevant parameters; 3) the type of GP used and the amount of GP added to the diet, and 4) the study that has a controlled treatment.

**Table 2:** PICO framework used in this meta-analysis.

PICO	Search strategy	Exclusion criteria
Population	Broiler chickens	Any chickens apart from broilers
Intervention	Grape pomace	Not in grape pomace
Comparators	Control group without grape pomace	
Outcomes	Growth performance, Carcass traits, and meat colour.	

The exclusion criteria applied in this study were as follows: 1) study without control group as one of the dietary treatments; 2) grey literature, narrative or systemic review and unpublished studies; 3) GP not included to the basal diet; 4) studies in which other conditions differed from the feeding standards of normal broilers; 5) a paper without animal ethical clearance

### Information, data extraction and analysis

Data on the surname of the first author, year of publication, country (South Africa, Spain Romania, Iraq, Turkey, Italy, Greece, Korea, Slovika, and Czech Republic), continents (Africa, Europe, South America, Asia), number of birds used, number of treatments, and moderator variables [strain (Ross and Cobb), offered form, inclusion levels (0-10, 11-20, 21-30, 31- 40 and >40) and feeding periods [(starter-grower, starter-finisher, grower-finisher] were retrieved from the 20 articles and used for the analysis (Table 3). All the analyses were done in OpenMEE software which is built in R-software. The standardized mean difference (SMD) and its 95 % confidence interval (CI) were selected as the effect scales to calculate the measured traits. The I^2^ value was used to estimate the heterogeneity among the different studies. When the heterogeneity test I^2^ was greater than 50 % and *P* < 0.05, statistical heterogeneity was considered to exist, and a random effects model was used for the meta-analysis (Harahap et al., 2022). However, subgroup analysis was not conducted for outcomes with < 3 comparisons because of low sample size (Ogbuewu et al., 2020). A funnel plot was used to determine publication bias among the studies and was determined by the symmetry and distribution of the funnel plot images (Xu et al., 2020). Sensitivity analysis was used to assess the stability of results with high heterogeneity and the impact of a single study on the overall analysis to determine whether the results are stable and reliable (Higgins et al., 2003; Zhan, 2010). The GP type was considered a subgroup factor, and a subgroup analysis was conducted to determine the source of heterogeneity.

**Table 3:** Description of articles used for this meta-analysis.

No	Author	Location	Continents	NT	Strain	1Age	Sex	IL	outcomes
1	Viveros et al. (2011)	Spain	Europe	4	Cobb	1-21	Male	51-60	1, 2,3
2	Pop et al. (2015)	Romania	Europe	3	Cobb	1-40	Mixed	11-20	1,2,3
3	Lichovnikova et al. (2015)	C. Republic	Europe	2	Ross	10-35	Mixed	11-20	2,3
4	Chamorro et al. (2015)	Spain	Europe	3	Cobb	1-21	Male	>60	1,2,3,
5	Pascariu et al. (2017)	Romania	Europe	6	Cobb	1-40	Mixed	11-20	1,2,3
6	Aditya et al. (2018)	Korea	Asia	4	Ross	1-28	Male	1-10	1,2,3,4,5,6,7
7	Kumanda et al. (2019)	South Africa	Africa	5	Cobb	14-42	Mixed	>60	1,2,3,4,5,8,9,10,11,12,13,14
8	Haščík et al. (2020)	Slovika	Europe	4	Ross	1-42	Mixed	21-30	4,5,7,8,13, 14
9	Nardoia et al. (2020)	Spain	Europe	6	Cobb	1-21	Male	51-60	1,2,3
10	Reyes et al. (2020)	Chile	South America	3	Ross	1-42	Male	>60	1,2,3,9,10,11
11	Turcu et al. (2020)	Romania	Europe	5	Cobb	14-22	Mixed	51-60	1,2,3,9,10,11
12	Abd AL-Kafar Sarhan et al. (2021)	Iraq	Asia	5	Ross	1-35	Mixed	>60	1,2,3
13	Gungor et al. (2021)	Turkey	Europe	4	Ross	1-21	Male	11-20	1,2,3
14	Romero et al. (2021)	Spain	Europe	5	Cobb	1-21	Male	>60	1,2,3,
15	Mavrommatis et al. (2021)	Greece	Europe	4	Ross	1-42	Mixed	21-30	1,2,3
16	Erinle et al. (2022)	Italy	Europe	3	Cobb	1-42	Mixed	21-30	1,2,3,8
17	Pascual et al. (2022)	Italy	Europe	3	Ross	1-42	Mixed	1-10	1,2,3,4,8
18	Giamouri et al. (2022)	Greece	Europe	4	Ross	1-42	Mixed	21-30	1,2,3,4,5,6,9,10, 11
19	Mauro et al. (2023)	Italy	Europe	3	Ross	1-42	Mixed	51-60	2
20	Themma et al. (2023)	South Africa	Africa	4	Ross	1-42	Mixed	41-60	1,2,3,5,6,8,9,10,11, 12,13,14

NT = Number of treatments; C. Republic = Czech republic, IL = Inclusion level in g/kg, 1 = average daily feed intake, 2 = average daily gain, 3 = Feed conversion ratio, 4 = carcass yield, 5 = liver, 6 = spleen, 7 = heart, 8 = breast, 9 = = L*, 10 = a*, 11 = b*, 12 = gizzard, 13 = thigh, 14 = drumstick.

1 Age = in days.

## Results

### Characteristics of articles used in the meta-analysis

Twenty (20) articles in this meta-analysis published from 2011 to 2022 in different countries from four continents (Europe, Asia, Africa, and South America) were used. In this, meta-analysis, grape pomace was included in the broiler diets as supplementation or replacement of synthetic antibiotics at inclusion levels ranging from 1 – 10, 11 – 20, 21 – 30, 31 – 40, 41 – 50, 51 – 60 and >60 g/kg in a studies with treatments group ranging from 3 to 4 and control as one of the treatments. Moreover, the inclusion level from 31 – 40 g/kg was removed from the analysis for having < 3 articles in their stratum. In the 20 articles included in this meta-analysis, ADFI, ADG and FCR results were performed from 46, 48, and 47 comparisons respectively on male or mixed-sex Cobb or Ross broiler strains. However, the broiler strains were fed a diet with GP from 1 – 42 days.

### Growth performance

**Fig 2:**
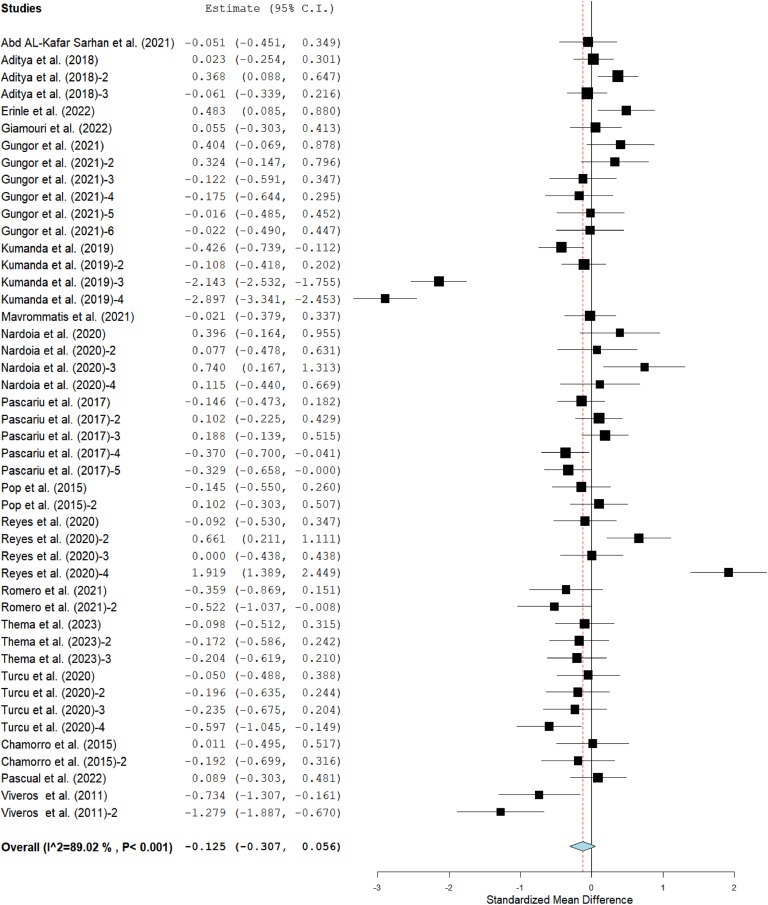
Forest plot of average daily feed intake (ADFI) of broiler chickens fed grape pomace. CI = confidence interval; I^2^ = Inconsistency index. The solid vertical line depicts a mean difference of zero (0) or no effect. Points to the left of the no effect line (zero) depict a decrease in ADFI and the opposite depicts an increase in ADF1. Individual square in the plot represents the mean effect size for each experiment, while the upper and lower 95 % CI for the effect size are the line that joined the squares. The dotted line with the diamond at the base showing the 95 % CI depicts the pooled estimation. I^2^ = inconsistency index is a measure of variance above chance among articles utilized in the analysis. Pooled estimation is considered significant when the line of no effect does not touch the diamond at the bottom of the forest plot (Koricheva et al., 2013).

**Fig 3:**
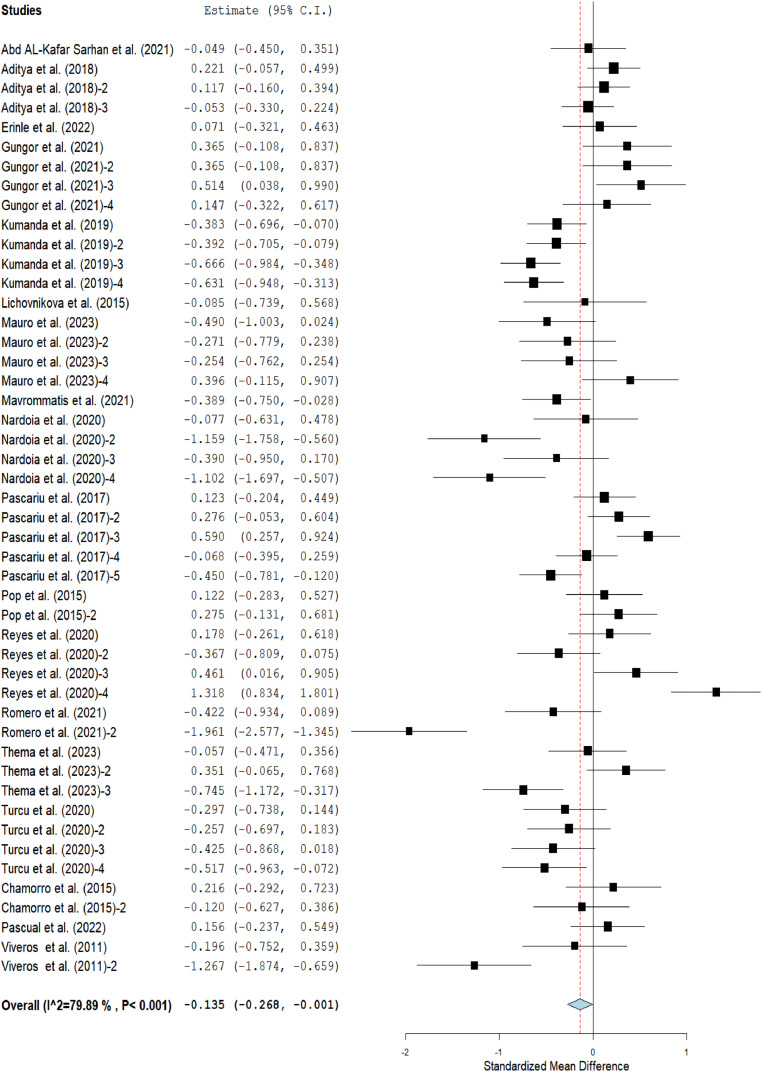
Forest plot of average daily gain (ADG) of broiler chickens fed grape pomace. CI = confidence interval; I^2^ = Inconsistency index. The solid vertical line depicts a mean difference of zero (0) or no effect. Points to the left of the no effect line (zero) depict a decrease in ADG and opposite depicts an increase in ADG. Individual square in the plot represents the mean effect size for each experiment, while the upper and lower 95 % CI for the effect size are the line that joined the squares. The dotted line with the diamond at the base showing the 95 % CI depicts the pooled estimation. I^2^ = inconsistency index is a measure of variance above chance among articles used in the analysis. Pooled estimation is considered significant when the line of no effect does not touch the diamond at the bottom of the forest plot (Koricheva et al., 2013).

**Fig 4:**
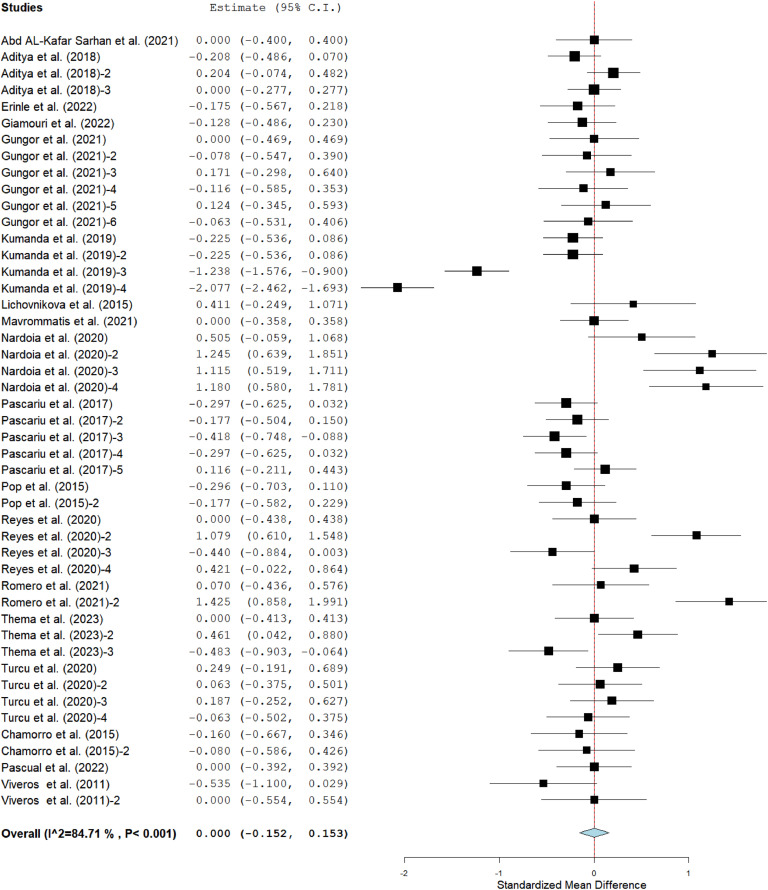
Forest plot of feed conversion ratio (FCR) of broiler chickens fed grape pomace. CI = confidence interval; I^2^ = Inconsistency index. The solid vertical line depicts a mean difference of zero (0) or no effect. Points to the left of the no effect line (zero) depict a decrease in FCR and opposite depicts an increase in FCR. Individual square in the plot represents the mean effect size for each experiment, while the upper and lower 95 % CI for the effect size are the line that joined the squares. The dotted line with the diamond at the base showing the 95 % CI depicts the pooled estimation. I^2^ = inconsistency index is a measure of variance above chance among articles utilized in the analysis. Pooled estimation is considered significant when the line of no effect does not touch the diamond at the bottom of the forest plot (Koricheva et al., 2013).

The meta-analysis results showed that the ADFI [SMD = −0.13; *P* < 0.001; *I^2^* = 89 %] and FCR [MD = 0.00; *P* < 0.001; *I^2^* = 85 %] of broilers fed grape pomace supplemented diets (GPBD) were comparable to those fed diet without GP in Figs. 2 and 4 respectively. Inversely, broilers chickens fed GPBD had lower (*p* < 0.05) ADG [SMD = −0.14; *P* < 0.001; I^2^ = 80 %] than those fed control group (Fig. 3).

### Carcass traits and internal organ weights

The effects of GPBD on carcass traits and internal organ weights of broiler chickens are presented in Table 4. In this table, the broilers fed diets with and without GP showed no difference in dressing percentage, breast, thigh, heart, and spleen weights. However, the weight of the drumstick and gizzard significantly improved on the broilers fed diet with GP. Conversely, broilers on GP-based diet (GPBD) had lower liver weight compared to the control group.

**Table 4:** Carcass traits and internal organ weights of broiler chickens supplemented with grape pomace.

Outcomes	SMD	95 % CI		SE	*p*-value	Heterogeneity			
		Lower	Upper			Q_M_	df	*P*-value	I^2^
Carcass traits									
Dressing percentage	−0.12	−0.33	0.09	0.11	0.001	52.4	11	<0.001	79.0
Breast	0.34	−0.30	1.06	0.35	0.272	389.79	11	<0.001	97.2
Drumstick	0.28	0.02	0.54	0.13	0.033	22.80	6	<0.001	73.7
Thigh	−0.19	−0.64	0.26	0.22	0.404	133.20	9	<0.001	93.2
Internal organs									
Gizzard	0.50	0.33	0.67	0.09	<0.001	18.6	9	0.030	51.7
Heart	−0.51	−1.12	0.13	0.33	0.120	107.2	5	<0.001	95.3
Liver	−0.39	−0.74	−0.05	0.17	0.023	175.9	13	<0.001	92.6
Spleen	−0.45	−1.12	0.23	0.34	0.192	153.6	6	<0.001	96.0

SMD = standardized mean, CI = confidence interval; SE = standard error; Q_M_ = coefficient of moderator; df = degree of freedom; *p* = probability difference; I^2^ = inconsistency index.

### Meat colour

In comparison with the control diets, pooled results show that broilers fed on grape pomace based diets (GPBD) had increased meat redness (Table 5). However, meat from broilers fed GPBD did not differ in terms of yellowness and lightness compared to meat from broiler fed control diets.

**Table 5:** Meat colour of broiler chickens supplemented with grape pomace.

Outcomes	SMD	95 % CI		SE	*p*-value	Heterogeneity			
		Lower	Upper			Q_M_	df	*P*-value	I^2^
Colour									
Redness (a*)	1.42	0.31	2.53	0.57	0.120	1598.8	19	<0.001	98.8
Yellowness (b*)	0.25	−0.30	0.80	0.28	0.375	611.5	19	<0.001	96.9
Lightness (L*)	0.04	−0.08	0.16	0.06	0.493	32.9	19	0.349	41.9

SMD = standardized mean, CI = confidence interval; SE = standard error; Q_M_ = coefficient of moderator; df = degree of freedom; *p* = probability difference; I^2^ = inconsistency index.

### Subgroup analyses

According to the subgroup analysis, the observed heterogeneity in ADFI, ADG, and FCR between studies was caused by the variations in different strains, supplementation inclusion level in the diet, broiler production age, and sex (Table 5, Table 6, Table 7). Mixed sex on Cobb broiler strain offered diets with GPBD had reduced ADFI while male Ross had comparable ADFI to that of control groups (Table 5). However, subgroup results indicate that inclusion levels at 1 – 10, 11 – 20, 21 – 30, 41 – 50, and >60 g/kg of GP and age of 1 – 21, 1 – 28, 1 – 42 and 22 – 42 days did not explain heterogeneity between the studies on ADFI. The broiler strains did not explain heterogeneity between the studies on ADG and FCR. Inclusion of GP in broiler diet at 1 – 10, 11 – 20, 41 – 50, 51 – 60, and >60 g/kg had comparable ADG when fed for 1 – 21, 1 – 28, 22 – 42 days to male broilers. Conversely, inclusion of GP in broiler diets at 21 – 30 g/kg had significantly decreased ADG when fed for 1 – 42 days to mixed-sex broiler chickens (Table 6). Results indicate that inclusion levels at 1 – 10 and 41 – 50 g/kg of GP significantly improved FCR, except those offered at 11 – 20, 21 – 30, 51 – 60, and >60 g/kg, which had statistically similar FCR (Table 7). Male broiler chickens fed GPBD had significantly poor FCR while the mixed sex ones had improved FCR compared to those fed controls. Nevertheless, the age of the broilers did not explain any heterogeneity between the studies on FCR (Table 8).

**Table 6:** Subgroup analyses of the effect of covariates of grape pomace on feed intake of broiler chickens.

Subgroup		Random Effects			Heterogeneity	
	Nc	SMD	95 % CI	*p-value*	I^2^ (%)	*p-value*
Strain						
Ross	20	0.13	−0.04, 0.29	0.147	72.1	<0.001
Cobb	26	−0.33	−0.61, −0.05	0.023	91.5	<0.001
Inclusion (g/kg)						
1-10	8	−0.09	−0.34, 0.15	0.463	77.3	<0.001
11-20	11	−0.02	−0.15, 0.11	0.793	4.9	0.400
21-30	10	0.02	−0.21, 0.24	0.895	63.7	0.003
41-50	3	−0.11	−0.34, 0.11	0.322	0.0	0.811
51-60	6	−0.59	−1.35, 0.17	0.127	93.2	<0.001
>60	8	−0.15	−0.11, 0.77	0.749	96.8	<0.001
Age (days)						
1-21	14	0.07	−0.30, 0.44	0.718	85.8	<0.001
1-28	3	0.11	−0.15, 0.37	0.403	60.8	0.080
1-42	27	−0.24	−0.50, 0.003	0.053	91.0	<0.001
22-42	2	−0.15	−0.48, 0.18	0.381	0.00	0.880
Sex						
Male	23	0.07	−0.14, 0.28	0.511	79.3	<0.001
Mixed	23	−0.31	−0.58, −0.03	0.028	91.9	<0.001

Nc = number of comparisons; SMD = standardized mean differences; CI = confident interval;.

I^2^ = Inconsistency index; *p* = probability difference.

**Table 7:** Subgroup analyses of the effect of covariates of grape pomace on weight gain of broiler chickens.

Subgroup			Random Effects			Heterogeneity	
	Nc		SMD	95 % CI	*p-value*	I^2^ (%)	*p-value*
Strain							
Ross	22		0.08	−0.09, 0.25	0.341	71.4	<.0001
Cobb	26		−0.32	−0.51, 0.13	<0.001	81.0	<0.001
Inclusion (g/kg)							
1-10	9		0.05	−0.18, 0.27	0.675	74.0	<0.001
11-20	9		0.14	−0.10, 0.36	0.211	57.1	0.017
21-30	11		−0.24	−0.40, −0.08	0.003	30.3	0.157
41-50	3		−0.33	−0.81, 0.15	0.178	75.3	0.180
> 60	8		−0.14	−0.69, 0.42	0.630	92.0	<0.001
Age (days)							
1-21	14	−0.27		−0.70, 0.17	0.226	89.4	<0.001
1-28	3	0.10		−0.07, 0.26	0.247	0.0	0.386
1-42	29	−0.15		−0.30, −0.01	0.031	70.1	<0.001
22-42	2	0.33		−0.31, 0.69	0.073	13.5	0.282
Sex							
Male	21	−0.13		−0.38, 0.13	0.338	85.5	<0.001
Mixed	27	−0.15		−0.29, −0.01	0.035	71.3	<0.001

Nc = number of comparisons; SMD = standardized mean differences; CI = confident interval; I^2^ = Inconsistency index; *p* = probability difference.

**Table 8:** Subgroup analyses of the effect of covariates of grape pomace on feed conversion ratio of broiler chickens.

Subgroup			Random Effects			Heterogeneity	
	Nc		SMD	95 % CI	*p-value*	I^2^ (%)	*p-value*
Strain							
Ross	21		0.05	−0.08, 0.18	0.463	54.6	0.001
Cobb	26		−0.04	−0.30, 0.22	0.786	89.8	< 0.001
Inclusion (g/kg)							
1-10	9		−0.14	−0.29, −0.001	0.049	37.8	0.116
11-20	11		−0.01	−0.14, 0.12	0.894	0	0.891
21-30	10		0.16	−0.06, 0.38	0.160	63.3	0.004
41-50	3		−0.29	−0.51, −0.06	0.012	0	0.539
51-60	6		0.09	−0.70, 0.90	0.821	93.9	<0.001
>60	8		0.03	−0.73, 0.80	0.932	95.7	<0.001
Age (days)							
1-21	14	0.32		−0.01, 0.64	0.056	81.6	<0.001
1-28	3	−0.001		−.0.23, 0.23	0.991	52.7	0.121
1-42	28	−0.14		−0.34, 0.05	0.156	86.0	<0.001
22-42	2	0.03		−0.30, 0.36	0.871	0	0.347
Sex							
Male	23	0.32		0.03, 0.43	0.023	77.5	<0.001
Mixed	24	−0.21		−0.41, −0.01	0.044	85.7	<0.001

Nc = number of comparisons; SMD = standardized mean differences; CI = confident interval; I^2^ = Inconsistency index; *p* = probability difference.

### Meta-regression and bias analysis

In Table 9, the results of meta-regression revealed that strain had effects on ADFI (*P* = 0.016; R^2^ = 10.9), and ADG (*P* = 0.004; R^2^ = 16.31). In contrast, there was not a significant relationship between ADFI, and ADG and moderators (inclusion, age and sex). Likewise, strain, inclusion level and age did not have any associations with FCR, whereas sex is a predictor for FCR (*P* = 0.004; R^2^ = 16.26).

**Table 9:** Meta‐regression of the associations between moderators and growth parameters.

Outcomes	Covariates	Intercept	Q_M_	Estimate	*p-*value	R^2^ (%)
Feed intake	Strain	0.13	5.79	0.37	0.016	10.91
	Inclusion	0.43	5.00	−0.16	0.603	0.00
	Age	−0.24	2.27	0.42	0.518	0.00
	Sex	−0.31	3.70	0.39	0.054	6.48
ADG	Strain	0.08	8.44	0.18	0.004	16.31
	Inclusion	−0.12	8.34	0.19	0.214	6.74
	Age	−0.15	2.88	0.21	0.411	0.00
	Sex	−0.15	0.05	0.22	0.828	0.00
FCR	Strain	0.06	0.38	0.28	0.540	0.00
	Inclusion	0.01	2.42	0.29	0.789	0.00
	Age	−0.14	6.12	0.25	0.106	6.80
	Sex	−0.21	8.31	0.23	0.004	16.26

ADG = Average daily gain; FCR = feed conversion ratio; Q_M_ = coefficient of moderators; *p* = probability difference; R^2^ = amount of heterogeneity accounted for by covariates.

**Fig 5:**
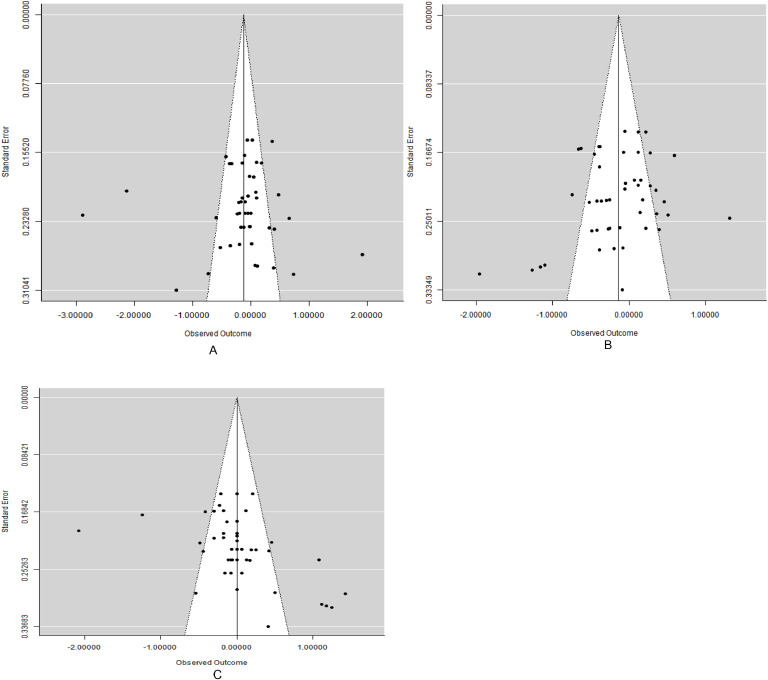
Funnel graphs of the effect of grape pomace supplemented diet fed to broiler chickens on Fig 5A, B and C.

Funnel graphs (Fig. 5) show a weak tendency for smaller studies to be associated with greater negative effects. The funnel graphs produced were symmetrical and Rosenberg’s fail-safe number (Nfs) was also employed to check for evidence of publication bias. The fail-safe numbers for the database are 147 (ADFI), 101(FCR), and 39 (ADG) which were 1.47, 1.06, and 2.44-fold higher than the thresholds of 100 (5 × 18 + 10), 95(5 × 17 + 10) and 95(5 × 17 + 10), respectively; therefore, declaration of the robustness of the mean effect size was required.

## Discussion

### Growth performance

Grape pomace are rich in bioactive compounds with anti-oxidative and antimicrobial properties that can enhance beneficial gut microbiota (*Bifidobacterium spp*. and *Lactobacillus spp*) and suppress harmful bacteria such as enterobacteriaceae family (Sinrod et al., 2023). Accordingly, GP could be used as a feed ingredient to promote poultry growth performance. Growth performance is a product of ADFI, which consequently affects both the FCR and the ADG in monogastric animals (Ogbuewu et al., 2022). However, Abu Hafsa and Ibrahim (2018) reported that GP has been used as a nutraceutical in broilers to boost growth and meat colour. In this meta-analysis, broiler chickens fed diets with GP had ADFI and FCR comparable to control birds, suggesting that GP could provides nutraceutical properties that can be used in poultry nutrition. Similar reports (Van Niekerk et al., 2020; Dupak et al., 2021) on broiler chickens found that feeding diets with or without GP had no effect on ADFI, and FCR. The observed similar ADFI and FCR in this study indicates similarity in the diets digestibility and utilisation of nutrients. The GP contains hydroxycinnamic acids which are known to increase the ratio of villus height to crypt depth thereby enhancing nutrients absorption and utilisation (Samuel et al., 2017). Additionally, these acids are also known to have antioxidants, anti-microbial, anti-viral, and anti-inflammatory properties (Lu et al., 2020) which directly and/or indirectly contribute to improving broiler’s performance. Also, polyphenol in GP such as flavonoids modify intestinal microflora by increasing the biodiversity degree of intestinal bacteria in broiler chicks (Viveros et al., 2011) leading to improved feed efficiency. However, this effects was not observed in the ADG results which was significantly low in ADG value in broilers in the GP group indicates low ability of the diet to support muscle accretion. This is linked with condense tannin in GP-containing diets which binds dietary amino acids (methionine) and digestive enzymes which are responsible for muscle accretion.

### Carcass traits and internal organ weights

Breast muscle is the most economically valuable muscle in modern broiler production and is used to measure the profitability of any poultry enterprise (Jonathan et al., 2020). However, in this meta-analysis, similar dressing percentage, breast, thigh, heart, and spleen weights of broiler chickens fed diets with or without GP could be due to a steady intake of lysine and methionine in the diets, an essential amino acids that induce the synthesis of the breast muscle relative to other muscles (Berri et al., 2008). In addition, GP contains flavonoids such as quercetin which are known to promote the production of protein in the muscles and also decrease the weight of immune organs (immunosuppressor) like spleen (Saeed et al., 2019) thereby promoting growth.

These current results confirm the findings reported by Jonathan et al. (2020) that the inclusion of GP in grower diets did not affect carcass trait of Hy-line Silver Brown cockerels. Likewise, similar heart and spleen weights were reported by Aditya et al. (2018) in broiler chickens fed a diet containing GP which is a sign of healthy broiler chickens. Spleen enlargement is an anatomical adaptation to fight against infectious (Jonathan et al., 2020). Heavier gizzards in the broiler chickens fed a diet with GP implies that a diet with GP is bulkier than a diet without GP (Control); hence this contributes to the development of the gizzard muscles due to grinding activity. Gizzard responds quickly to the change of coarseness of the diet and assists in reducing particle size thereby regulating feed flow (Svihus, 2011). However, in this study further investigation is warranted due to fewer published data on the gizzard weight of broilers fed GP-containing diets. Likewise, heavier drumstick muscles and lack of difference in breast weight muscles from broiler chickens fed diets with and without GP in their diets has unknown aetiology. However, this finding is difficult to explain and requires further investigation. Small weight liver in broilers fed diets with GP could be a sign of less activity of the liver to detoxify anti-nutritional factors (ANFs) present in GP, a result contradicts the findings of Kara et al. (2016) in laying quails and Giamouri et al. (2022) in broiler chickens when GP is incorporated in their diets. This result implies that the GP used in this study has ANFs within the normal range of broiler chickens as shown by similar ADFI, ADG, and FCR.

### Meat colour

Colour of the meat influences customer purchasing decisions since it is the major sign of freshness (Sabow et al., 2022). In this meta-analysis, GP significantly increased redness of the broiler meat which could be linked to the presence of anthocyanins pigmentation in grapes which interacted with enzymes involved in colour formation. Fletcher (1999) reported that diet is one of the factors that influence the colour of breast meat. These results imply that anthocyanins in GP have a role as an antioxidant to improve the red colour in the meat. Similar findings were reported by Bennato et al. (2020) in Ross 308 meat when offered diets with GP. In contrast, no differences were observed in lightness and yellowness of the meat of broiler chickens fed diets with and without GP, a findings that are consistent with earlier literature on the effects of feeding diets with GP on meat colour in broiler chickens (Kasapidou et al., 2016) and Japanese quails (Sabow et al., 2022).

### Subgroup analyses

#### Strain

Strain is a significant predictor in FI, ADG, and FCR across included studies in this meta-analysis which explained the small effects of strains as a moderator. Cobb had significantly lower FI compared to Ross when fed diet with GP implies that Ross strain can overcome negative effects of GP on ADFI better than Cobb strain. These findings are consistent with the findings of other researchers (Manyeula et al., 2025b and Ogbuewu et al., 2024) who reported difference FI in different strains of broiler chickens when fed similar diets. It can be deduced that Cobb efficiently utilized diet with GP to meet its protein and energy requirements at lower intake. Ferket and Gernat (2006) reported that birds consume feed to fulfil their protein and energy requirements. Similar ADG and FCR on Cobb and Ross on diet with and without GP indicates the ability of these strains to utilize GP at the same extent, a result in harmony with Manyeula et al. (2025a), who reported similar results in ADG of Cobb and Ross supplemented with rapeseed meal. This could be attributed to the genetic differences and feed conversion potential of the two strains. Li et al. (2024) reported that FCR is associated with genetic which play a role in digestibility, metabolism, stress response and energy homeostasis. Further studies are needed to explore the factors that support lower ADFI with enhanced ADG in Cobb broiler chickens.

#### Inclusion level

Broilers consume a diet to fill their gut if not limited by dietary toxicities, environment, management practices, and disease factors. Grape pomace is known to contain condense tannins (Onache et al., 2022) which reduce ADFI when consumed indirectly or directly. This tannin also is influenced by the level of GP inclusion which determines the levels of toxicity. In this meta-analysis, it was expected, therefore, that higher inclusion of GP in broiler chicken diets might reduce ADFI but this was not the case. Indeed, higher inclusion levels (>60 g/kg) did not affect ADFI in broiler chickens. This suggests that GP inclusion at > 60 g/kg does not negatively affect palatability and functional properties of the diet hence similar ADFI. The reduced ADG of broilers fed diet with GP at 21 – 30 g/kg is an indication of poor nutrient utilization by broilers chickens. Conversely, inclusion of GP in broiler diets at 1 – 10, 11 – 20, 41 – 50, 51 – 60 and >60 g/kg had comparable ADG with broiler chickens fed diets with or without GP. This suggests similar nutrient utilization of broiler chickens fed diets with or without GP hence GP could be used as an alternative without affecting ADG of broiler chickens. In contrast, a high inclusion level of GP was reported to reduce the growth performance of broiler chickens (Viveros et al., 2011). In this meta-analysis, inclusion levels were another significant predictor of FCR. However, subgroup analysis showed that feeding the broilers diets with GP at 1 – 10 and 51 – 60 g/kg improved FCR, indicating a proper inclusion level that could be used to improve absorptive capacity and functions of the small intestine for better FCR. The higher FCR observed in the broiler’s chickens fed diet with GP at 1-10 and 51-60 g/kg may be attributed to the fact that some polyphenolic bio-active compounds in GP decreased intestinal absorption of nutrients. This occurrence may have positively influenced ADFI hence weight gain. Contrary to the previous findings, broiler chickens fed diets with GP at 11-20, 21-30, 41-50, 51-60 and > 60 g/kg had comparable FCR with those fed diets without GP, implying similar absorptive capacity and functioning of the small intestines. Similarly, Brenes et al. (2008) and Chamorro et al. (2015) reported that inclusion of up to 60 g/kg in broiler diets did not affect broiler chicken performance. The comparable FCR in broiler chickens fed diets with and without GP could be due to the broilers benefiting from polyphenols bio-active compounds present in GP that promote intestinal absorption.

#### Age

Subgroup analysis demonstrated that age is not a limiting factor in this study In contrast, Chamorro et al. (2013) reported a decreased growth performance and diet digestibility in broiler chickens fed diets with GP from 1 to 21 days. A reduced ADG is commonly influenced by ADFI. However, in this case, significantly lower ADG in broiler chickens reared on diets with GP for 1-42 days could be attributed to the bird’s failure to cope with GP feeding for a long time. Moreover, part of this reduction could be due to gut microbiota converting condensed tannins into other bio-active metabolites which negatively affect gut mucosa and microbiota composition (Redondo et al., 2022).

#### Sex

Subgroup results demonstrated that sex is a limiting factor in ADFI and ADG which can lead to varying growth performance results. The similar ADFI and ADG in male broiler chickens compared with mixed sex (males and females) in this meta-analysis suggests that males utilized GP-containing diets better than mixed sex. This could be due to feeder space and competition. It is known that the males will have a high ADFI and ADG under mixed-sex rearing due to their dominance (England et al., 2023). In contrast with current findings, Dupak et al. (2021), reported that male broiler chickens had higher ADG compared to females when offered a diet containing 450 mg/kg GP. The FCR is influenced by animal size, feed quality, feeding rate, husbandry practices, and age (Lie et al., 2014) and it determines the performance and profitability of the chicken enterprise (Ogbuewu et al., 2023). Furthermore, it also measures feed efficiency. However, in this subgroup analysis, the males had poor FCR when fed diets with GP. Inversely, mixed-sex had better FCR when fed diets with GP, indicating that mixed-sex is more efficient compared to males when fed diets with GP and could be more profitable when used on the boiler production business.

### Meta-regression and bias analysis

The meta regression results showed evidence of significant relationships between broiler strains on FI and ADG, implying that these parameters acted jointly with strains. This finding agreed with the meta-analysis by Manyeula et al. (2025a), who reported relationship between broiler strains and FI. Likewise, Zhang et al. (2020) and Olusegun et al. (2020) reported that strains significantly influence the growth performance parameters. The significant relationship between strains and ADFI and ADG could be related to genetic makeup of the birds. Also, in this meta-analysis, sex is a predictor for FCR in broiler chickens fed diets with GP, a result confirmed by Benyi et al. (2015), who reported a relationship between sex and FCR in commercial broiler strains. Udeh et al. (2015) also found significant genotype X sex effects on ADFI, ADG and FCR of broilers. The lack of a significant relationship between inclusion levels, age, and sex on ADFI and ADG and strain, inclusion and age on FCR implies that ADFI, ADG and FCR do not depend on the parameters in question. Further research to determine the profitable inclusion levels of GP that optimized the growth performance parameters in broiler chickens is needed, as such data is limited in the literature.

## Conclusions and future research direction

The pooled results showed that broiler chickens fed diets without GP performed better in terms of liver weight, ADFI, ADG, and FCR than those fed GP-based diets. On the other hand, dressing percentage, breast, thigh, heart, and spleen weights were not affected in broiler chickens by GP. The pooled results indicated that GP-based diets improved meat redness and weights of drumstick in broiler chickens. Subgroup analysis showed that Cobb strain at levels of 1 to > 60 g/kg of GP had similar ADG and FCR, whereas Ross strain had comparable ADFI, ADG and FCR. Additionally, broiler chickens fed diets with GP at 1-10, 11-20, 21-30, 41-50, 51-60 and >60 g/kg did not affect ADG. Moreover, inclusions at 1-10 and 41-50 g/kg improved FCR in broiler chickens fed GP-based diets while other inclusions did not affect FCR. Furthermore, the inclusion of GP in the broiler diets did not affect ADFI. Feeding broiler chickens for 1 to 42 days did not affect ADFI and FCR of broilers fed diets with GP. Lastly, mixed sex had decreased ADFI and ADG with improved FCR while males were not affected by the inclusion of GP. Meta-regression showed that strain explained most of the sources of heterogeneity in the ADFI and ADG while sex explained variation in FCR. The effect of inclusion of GP at 31-40 g/kg as a moderator on the impact of GP inclusion levels on growth performance, carcass and meat colour of broiler chickens could not be determined in the present study due to low sample size, and future studies is needed on the effects of different inclusion levels of GP in the broiler diets. Therefore, the use of biotechnological methods such as probiotics and enzymes to improve nutritional qualities of GP as to maximise its utilisation in poultry feeding is recommended such information is lacking in the literature

## Limitations and strengths of the meta‑analysis

This meta-analysis of feeding diets with GP on broiler chickens has a certain limitations including small number of articles and experimental data that might have impacted on the accuracy of the results. For example, regression and subgroup analysis on the inclusion levels of GP in the broiler chickens at 31 - 40 g/kg was not performed due to fewer studies obtained. Furthermore, in the 20 articles used in this study, variations in the analytical methods, soil types used for planting grapes and study locations could have influenced the validity of the results. Despite these limitations, pooling results on the topic to increase statistical power, identify research gaps, resolve disputes, and generate new insights is one of the strengths of this study. This work contributes to the knowledge of incorporating GP on the diets of broiler chickens and setting the guidelines for standardized experimental designs in future trials.

## Declaration of competing interest

There is no conflict of interest associated with publishing this manuscript.
